# Allogeneic Bone-Marrow Mesenchymal Stem Cell with Moldable Cryogel for Craniofacial Bone Regeneration

**DOI:** 10.3390/jpm11121326

**Published:** 2021-12-07

**Authors:** Cheng-Feng Chu, Shih-Hsuan Mao, Victor Bong-Hang Shyu, Chih-Hao Chen, Chien-Tzung Chen

**Affiliations:** 1 Department of Plastic and Reconstructive Surgery, Keelung Chang Gung Memorial Hospital, Keelung 204, Taiwan; cfjosephchu@gmail.com (C.-F.C.); vbshyu@gmail.com (V.B.-H.S.); chchen5027@gmail.com (C.-H.C.); 2 Department of Plastic and Reconstructive Surgery, College of Medicine, Chang Gung University, Linkou Chang Gung Memorial Hospital, Craniofacial Research Center, Taoyuan 333, Taiwan; ray.sh.mao@gmail.com

**Keywords:** allogeneic cellular transplantation, bone-marrow mesenchymal stem cells, bone regeneration, cryogel

## Abstract

Allogeneic bone-marrow mesenchymal stem cells (BMSCs) can promote bone regeneration and substitute for autologous BMSCs if autologous sources are unavailable, but the efficacy of bone regeneration by allogeneic BMSCs is still inconsistent. A Lewis rat cranium defect model was used to investigate the efficacy of bone regeneration between autologous and allogeneic BMSCs in gelatin-nanohydroxyapatite cryogel scaffolds. BMSCs from Wistar rats served as the allogeneic cell lineage. The full-thickness cranium defects were treated by either blank control, cryogel only, allogeneic BMSC-seeded cryogel, or autologous BMSC-seeded cryogel (*n* = 5). Bone regeneration was monitored by micro-computed tomography and examined histologically at week 12. In addition, we assessed the immune responses in vitro by mixed lymphocyte reaction (MLR) assay and CD_4_^+^ immunochemistry staining ex vivo. The MLR showed that allogeneic BSMCs elicited a weak immune response on day 14 that progressively attenuated by day 28. In vivo, the bone regeneration in allogeneic BMSCs was inferior at week 4, but progressively matched the autologous BMSCs by week 12. Our results suggest that allogeneic BMSCs can serve as an alternative source for bone regeneration.

## 1. Introduction

Restoring a bone defect remains a challenging clinical problem. Surgeons can reconstruct defects with autologous bone tissue transfer, but the need for a donor site increases overall morbidity and patient discomfort. Tissue engineering provides a potential alternative to bone regeneration by incubating cells, scaffold, and growth factors ex vivo, which alleviates the disadvantages of conventional clinical modalities [1,2].

Among the aforementioned critical factors, cells of adequate quality and quantity are indispensable to successful tissue engineering. Mesenchymal stem cells (MSCs) of various origins, such as adipose, bone marrow, dental pulp, and umbilical cord blood, have shown osteogenic potential [3,4]. Of these, bone-marrow mesenchymal stem cells (BMSCs) demonstrate a superior osteogenic effect compared to other sources [5]. Furthermore, the application of BMSCs has already been demonstrated in clinical settings [6,7]. However, they can be impractical because the quality and quantity of MSCs degenerate with the donor’s age and underlying medical conditions, such as diabetic mellitus or immunocompromised status [8,9]. Additionally, cell preparation is time-consuming and prevents prompt usage, while the use of autologous donor sites can add morbidity to patients. Thus, allogeneic MSCs may serve as an alternative cell source, with the potential to be expanded and preserved in advance, improving accessibility [10].

However, immune rejection is inevitable when it comes to allogeneic transplantation. Despite the immunological privilege of BMSCs, reports on the osteogenic potential of allogeneic BMSCs remain inconsistent throughout the literature. Allogeneic BMSCs have been reported to promote bone regeneration equivalent to autologous BMSCs in long bone and craniofacial bone defect models [10,11,12,13,14]. Alternatively, Coathup et al. demonstrated that allogeneic BMSCs failed to regenerate tibial defects, and positive mixed lymphocyte reaction assays suggested an immune response to the allogeneic cells [15]. Likewise, allogeneic MSCs in subcutaneous implants elicited an acute rejection in immunocompetent mice, and both T cell- and B cell-mediated immune responses precluded osteogenic processes [16,17]. This existing controversy implies that immune rejection may impede the clinical application of allogeneic BMSCs in bone regeneration.

While there has been extensive discussion regarding long bone regeneration in the literature, the reconstructive demands of craniofacial bones differ from long bones [18]. Instead of load-bearing ability, they require more morphological shaping. In previous studies of allogeneic bone regeneration, tricalcium phosphate (TCP) was employed for load-bearing areas, such as the mandible or tibia [10,11,12,13]. However, its characteristic of fast degradation may compromise the reconstructive outcomes in craniofacial bones because of the high demand to maintain the three-dimensional structures. Conversely, nano-hydroxyapatite (n-HAP) comprises a majority of the inorganic compounds in the natural bone extracellular matrix, and its slow resorption has been suggested in bone reconstruction for non-load-bearing regions [19]. Our previous results also demonstrated that the gelatin-nHAP served as an ideal scaffold for bone regeneration based upon its suitable mechanical characteristics and excellent biocompatibility [20]. The nHAP-gelatin cryogel behaves like a sponge and includes the advantages of plasticity and tailorability, which are critical characteristics in reconstructing craniofacial regions that require detailed design, such as calvarium.

This study aims to investigate the potential application of allogeneic BMSCs in bone regeneration via a rat cranium defect model and compare the outcomes of bone regeneration between allogeneic and autologous BMSCs seeded in gelatin-nHAP cryogels.

## 2. Materials and Methods

### 2.1. Extraction and Expansion of BMSCs from Rats

BMSCs were extracted from the femur bone marrow of 10- to 15-week Lewis or Wistar rats. To evaluate the cryogel degradation in vivo and the osteogenic process without potential immune perturbation, we used nude mice as the experiment animal. Briefly, the femur bone was removed, followed by repeated irrigation of the bone marrow cavity with sterile culture low glucose Dulbecco’s Modified Eagle Medium. Collected bone marrow and cells were filtered via a 100 μm cell strainer, centrifuged at 1000 rpm for 10 min, and then transferred onto a 10 cm Petri dish. The cells were cultured in low glucose Dulbecco’s Modified Eagle Medium (DMEM) with 10% fetal bovine serum (FBS), 1% penicillin, and streptomycin. Culture medium was replaced every 3 to 4 days. After 7 to 10 days, the colonies were subcultured. Passage 3 to 5 were used in the following experiments.

### 2.2. Flow Cytometry

The colonies were washed twice by PBS and trypsinized before centrifuging. A total amount of 1 × 10^6^ cells was used for flow cytometry (BECTON DICKINSON FACSAria IIu). The single-cell suspension was incubated with anti-CD90.1 (PerCP Mouse Anti-Rat CD90/Mouse CD90.1, BD, Franklin Lakes, NJ, USA) and anti-CD45 (Cy™5 Mouse Anti-Rat CD45, BD) for 1 h at 4 °C, followed by staining with fluorescent secondary antibodies for 30–45 min at 4 °C. TNAP^+^ BMSCs were sorted by incubating with anti-TNAP (anti-alkaline phosphatase, tissue non-specific antibody, Abcam, Cambridge, UK) for 30 min at 22 °C, followed by conjugated antibody (Goat polyclonal Secondary Antibody to Rabbit IgG, Abcam).

### 2.3. Mixed Lymphocyte Reaction Assay (MLR)

Lymphocytes were obtained from the spleen of Lewis rats. The removed spleen was grounded in 1 mL RPMI-1640 with 1% 2-Mercaptoethanol and 1% amino acid, then filtered by a 100 μm cell strainer. The solution was centrifuged at 1800 rpm for 3 min and the upper solution was discarded. Next, the pellet was mixed with 5 mL of Ammonium-Chloride-Potassium (ACK) solution followed by another period of centrifuging (1800 rpm for 3 min). Meanwhile, the responder cells, spleen-derived lymphocytes, were stained with CFSE (carboxyfluorescein diacetate succinimidyl ester), a marker for cell proliferation and decrease in intensity of CFSE due to cell division in the presence of an immune response. The responder cells were then mixed with the stimulator cells from Wistar rat-derived BMSCs in ratios of 1:1 and 1:3. Concanavalin A (ConA), a mitogen that induces T cell immune reaction, was used as a positive control. The response between responder cells and stimulator cells was assayed by flow cytometry.

### 2.4. Calcium (Ca^2+^) Stain and Alkaline Phosphatase (ALP) Stain

For calcium staining, the BMSCs were fixed by 1% formaldehyde for 20 min and stained with Alizarin-Red solution (Sigma, St. Louis, MO, USA) for 3 min. For alkaline phosphatase stain (ALP), a fixative solution (citrate working solution: acetone in a ratio of 2:3) was added to the BMSCs and staining was done with ALP stain solution (Sigma, 85L2-1KT) and Mayer’s hematoxylin solution. After staining, the BMSCs were rinsed with distilled water and documented by microscope.

### 2.5. Cryogel Preparation

The cryogel preparation was described previously [21]. Briefly, two grams of gelatin and one gram of pre-weighed nanohydroxyapatite (nHAP) were dissolved in a 20 mL 2-(N-morpholino)ethanesulfonic acid (MES) buffer solution (pH = 6.5) at 70 °C, then mixed at an equal volume ratio with dissolved EDC in a 10 mL MES buffer (pH = 6.5) at 0.02 M concentration. Next, the nHAP-gelatin solution was suspended in a 3 mL syringe and evenly stirred. The solution was then transferred to a −17 °C cooling bath for 16–24 h to complete the cryogelation process. Finally, the syringe mold was removed, and cryogel scaffolds were cut into cylinder-shaped discs of 1 mm thickness and 4 mm diameter.

### 2.6. Preparation of Cell-Seeded Cryogel Scaffolds

A total of 7.5 × 10^5^ cells, autologous or allogeneic BSMCs, were seeded onto each cryogel and incubated in an osteoinductive medium (DEME/F12 with 10 mM of beta-glycerophosphate, 5 mg/mL dexamethasone phosphate, and ascorbate sodium) 7 days before implantation in vivo.

### 2.7. Live Dead Cell Viability Assays

The Live Dead cell viability assay kit (Sigma) was used to verify the cell viability of the cell-seeded scaffolds under the manufacturer’s protocol. The Live/Dead staining solution was prepared with 3 μL of 4 mM calcein-AM (excitation 494 nm and emission 517 nm) and 5 μL of 2 mM ethidium homodimer-1 (EthD-1) (excitation 528 nm and emission 617 nm) in 10 mL PBS, respectively. All samples were incubated in 300 μL of staining solution for 15 min at 37 °C and imaged under a microscope.

### 2.8. Scanning Electron Microscope (SEM)

After 7 days of incubation, the cell-seeded scaffolds were fixed with 2.5% glutaraldehyde for 24 h at room temperature. After thoroughly washing with 0.1 M PBS (pH = 7.4), the samples were dehydrated in ethanol in a sequential manner (50%, 70%, 80%, 90% and 95%) for 15 min each, immersed in 99.5% ethanol for 20 min, dried in a critical point dryer (Leica EM CPD300, Wetzlar, Germany), and observed by SEM (JEOL ISM-5410, Tokyo, Japan) after gold coating.

### 2.9. Subcutaneous Implantation and Degradation Profiles

The Institutional Animal Care and Use Committee of Chang Gung Hospital approved the animal protocol based on the standards of the Association for Assessment and Accreditation of Laboratory Animal Care. Briefly, the dorsal skin of nude mice was carefully incised, followed by implantation of cell-seeded scaffolds. The wound was closed primarily and dressed with antibiotic ointment to avoid infection. The constructs were retrieved at week 2, 4, 6, and 8 and documented by photography and histological examinations.

### 2.10. Transplantation of Allogeneic BMSCs onto Cranium Bone Defect

Eight-week-old Lewis rats were used in the experiment. Briefly, the animals were anesthetized with isoflurane and cranial hair was removed by a hair clipper. Next, under sterile conditions, the cranium was exposed through a longitudinal cut over the scalp skin and three 4 mm, round, full-thickness defects were burred out. Each defect was designated as control, cryogel only, and cryogel seeded with autologous or allogeneic BMSCs. The wounds were then closed primarily. Postoperative care included analgesia with Ketoprofen (1 mL/kg) and infection control with Ampolin (1 mL/kg) for 3 days.

### 2.11. Micro-CT Imaging

Calvarial bone regeneration was assessed by micro-computed tomography (CT) (Mediso, Budapest, Hungary, nanoScan^®^ SPECT/CT). Images were taken under general anesthesia on day 0, week 4, 8 and 12 after implantation. The volume of bone regeneration was calculated through serial sagittal views by ITK-SNAP software (ITK-SNAP 3.8.0 version). A single operator performed the calculation to minimize calculation bias [22].

### 2.12. Histological Examinations

Animals were euthanized on week 12 after taking CT images. The region of interest was dissected out and then fixed with 10% formaldehyde and dehydrated with alcohol. The tissue was embedded into paraffin and cut into sections. Sections were transferred to alcoholic gradient dehydration and rehydrated for immunohistochemistry. The section was boiled in 10 mM sodium citrate for 20 min and rinsed in 10% H_2_O_2_ for 10 min. Histology included hematoxylin and eosin (H&E) and Masson’s Trichrome stain.

### 2.13. Immunohistochemistry Stain

Slides were baked at 60 °C for 15 min twice, followed by dewaxing in xylene for 10 min three times, and then rehydrating with 100%, 95%, 90% and 70% alcohol for 10 min consecutively. The antigens were retrieved by proteinase K (1:100) for 5 min. Three percent H_2_O_2_ was then administered for 15 min and washed by PBST for 5 min, followed by blocking with 3% BSA solution for 30 min. The slides were incubated with CD_4_^+^ primary antibody (Taiclone, Taipei, Taiwan), 1:200, for 60 min and washed with PBST for 5 min three times. Secondary antibody (Arigo, Hsinchu City, Taiwan), 1:1000, was then applied for 30 min and washed with PBST for 5 min three times. DAB (3,3′-Diaminobenzidine) was then added and washed with PBST for 10 min. The slides were then stained for cell nuclei for 15 min and rinsed under tap water for 20 min.

### 2.14. Statistical Evaluation

All data are presented as mean ± standard deviation (sd). The sample size was estimated to achieve a power of 0.8 and an α-level = 0.05 using analysis of variance (ANOVA). Two-tailed nonparametric Kruskal–Wallis tests with Dunn’s multiple comparison post hoc test were performed among multiple groups using SPSS software (SPSS Inc., Chicago, IL, USA). Statistical significance is considered as *p*-value < 0.05.

## 3. Results

### 3.1. Characteristic and Osteogenic Potential of BMSC

**Figure 1:**
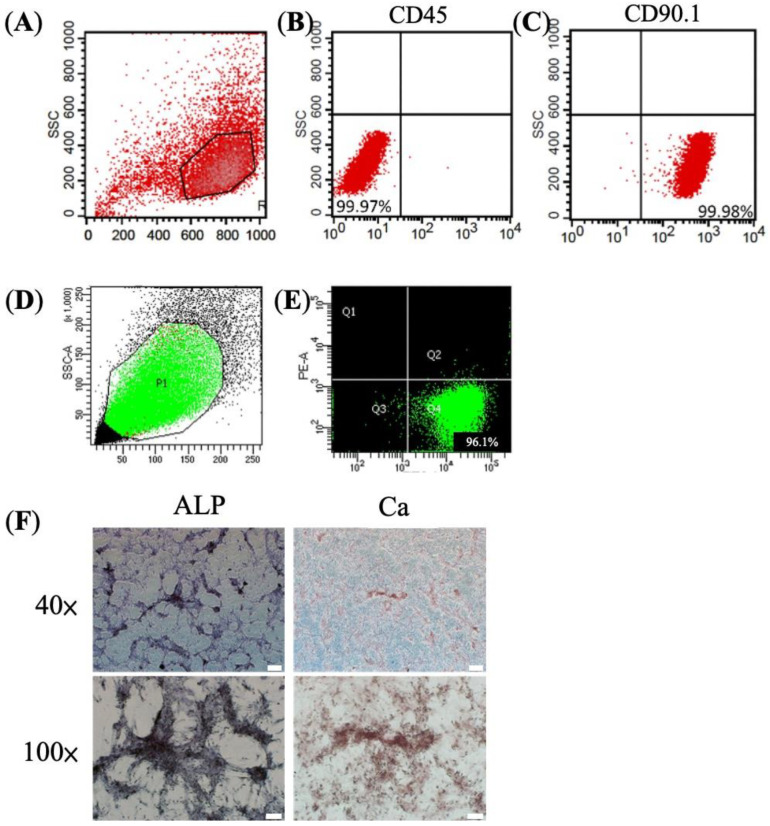
Characteristic and osteogenic differentiation of BMSCs in vitro. (**A**) Flowcytometry of BMSCs sorted from rat’s bone marrow (**B**) with negative CD45 and (**C**) positive CD90.1. (**D**,**E**) TNAP^+^ BMSC population sorted from BMSC colonies for their increased osteogenic differentiation potential. (**F**) Osteogenic differentiation demonstrated by positive ALP staining and calcium (Ca) deposition staining at day 28. Scale bar = 400 μm.

From the selected colonies, cells were identified as BMSCs by flow cytometry, which included positive CD 90.1 and negative CD 45 markers [3] (Figure 1A–C). We further sorted for TNAP^+^ BMSC population, which possesses increased osteogenic potential [23] (Figure 1D,E). The TNAP^+^ BMSCs showed strong ALP production and calcium deposit after 28 days of incubation, suggesting osteogenic commitment and phenotype (Figure 1F).

### 3.2. Mixed Lymphocyte Reaction (MLR) Assay

**Figure 2:**
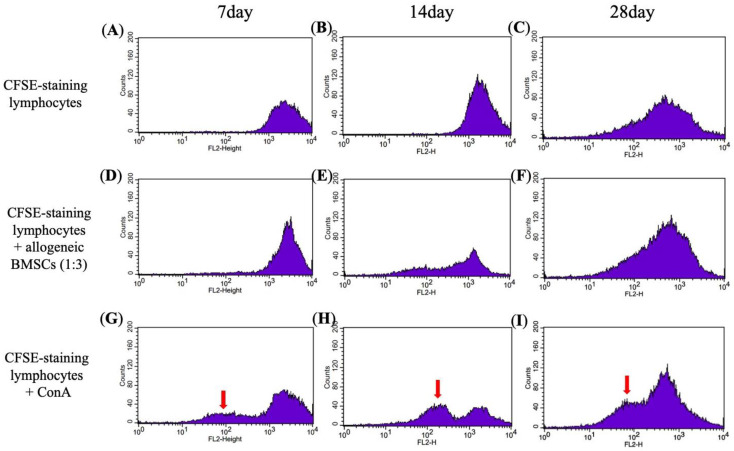
Assay of immune rejection in vitro by mixed lymphocyte reaction assays (MLR). CFSE-staining lymphocytes as a negative control at (**A**) day 7, (**B**) day 14, and (**C**) day 28. CFSE-staining lymphocytes were incubated with allogeneic BMSCs in a ratio of 1:3 at (**D**) day 7, (**E**) day 14, and (**F**) day 28. CFSE-staining lymphocytes stimulated with ConA as a positive control at (**G**) day 7, (**H**) day 14, and (**I**) day 28. A left-shift peak (red arrow) indicates lymphocyte proliferation, suggesting an immune response to external stimuli.

The immune response in vitro was examined via a mixed lymphocyte reaction (MLR) assay. By day 7, the allogeneic BMSCs did not activate the CFSE-staining lymphocytes. However, a declination of fluorescent and left shifting in peaks in the positive control of CFSE-staining lymphocytes and ConA was noted, indicating a positive immune response (Figure 2D,G). On day 14, the peak shifted left in CFSE-staining lymphocytes co-cultured with allogeneic BMSCs (Figure 2E), but to a lesser degree than the positive control (Figure 2H), suggesting a minor immune response. On day 28, the immune response was attenuated (Figure 2C,F). Thus, the results indicate that allogeneic BMSCs likely elicit an early immune response between day 7 and 28.

### 3.3. In Vitro Culture and In Vivo Degradation of Cryogels

**Figure 3:**
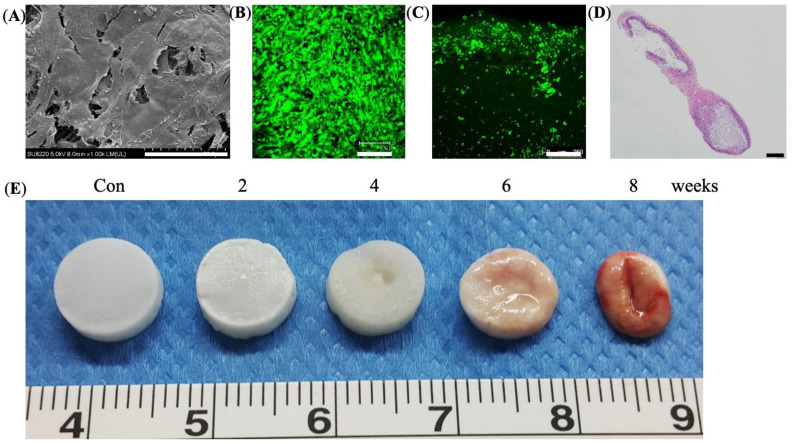
In vitro cultivation and subcutaneous implantation of BMSC-seeded cryogel. The BMSCs were seeded on the cryogel and incubated in vitro for 7 days before implantation. (**A**) SEM of BMSC-seeded cryogel (Scale bar = 50 μm), (**B**) live/dead assay for cell-seeded cryogel, top view, Scale bar = 250 μm, and (**C**) cross-section view. BMSC-seeded cryogels were implanted into the back of nude mice, Scale bar = 300 μm. (**D**) H&E stain of the bone regeneration in subcutaneous BMSC-seeded cryogel in nude mice at week 8, Scale bar = 400 μm. (**E**) In vivo degradation of the acellular cryogel at different time point. Con = control, cryogel before implantation.

SEM revealed that seeded cells were distributed on the scaffold and produced extra-cellular matrix (Figure 3A). Next, the live/dead cell assay verified that live cells were dispersed on the surface and infiltrated 0.75 to 1.00 mm in depth (Figure 3B,C). The histology revealed bone regeneration of implanted BMSC-seeded cryogel scaffolds subcutaneously at week 8 (Figure 3D). Finally, by the in vivo degradation assay, the cryogel scaffold (unseeded) gradually degraded throughout the 8-week observation period (Figure 3E).

### 3.4. Animal Study of Bone Regeneration

#### 3.4.1. Micro-Computed Tomography (Micro-CT)

**Figure 4:**
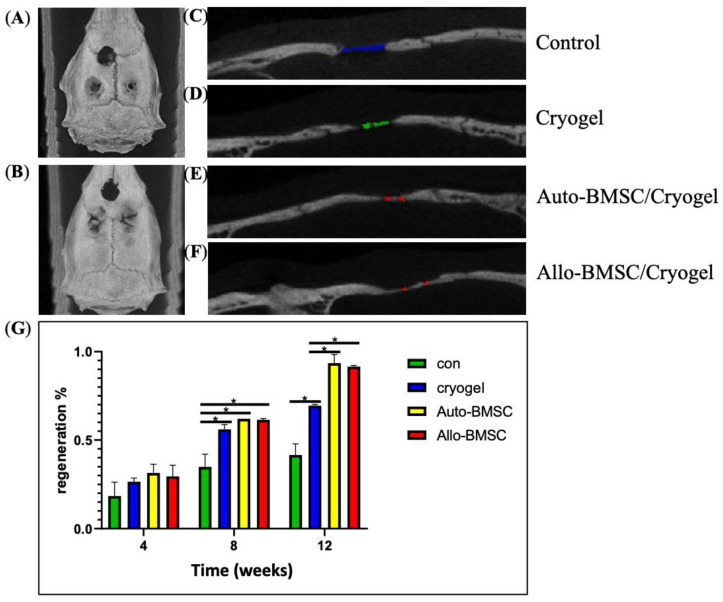
Bone regeneration in rat calvarial-defect model at week 12 by micro-CT. Upper defect: control; lower left: cryogel; lower right: cryogel and BMSC. (**A**) Axial view of autologous BMSC at lower right defect. (**B**) Axial view of allogeneic BMSC at lower right defect. (**C**) to (**F**) Sagittal view of skull defects. Defects marked with colors. (**C**) Control. (**D**) Cryogel. (**E**) Cryogel and autologous BMSC. (**F**) Cryogel and allogeneic BMSC. (**G**) Percentage of bone regeneration in bar graph (mean and SD) measured via micro-CT. * *p* < 0.05.

To evaluate osteogenic efficacy in vivo, we applied a cranium defect model. The minimum sample number for each group was 4 (ANOVA, power of 0.8 and α-level = 0.05). The defects were grouped into blank control, cryogel scaffold, and BMSC-seeded cryogel scaffold groups (Figure 4A,B) (*n* = 5 for each group). Micro-computed tomography (micro-CT) axial and sagittal views at week 12 show that both allogeneic and autologous BMSC-seeded cryogel scaffolds demonstrate higher radiopacity and bone regeneration than the cryogel group and control group (Figure 4A–F). At week 4, there was no statistical difference in bone regeneration among groups (Figure 4G). Notably, the allogeneic BMSCs showed less regeneration than autologous BMSCs (30.8 ± 5.4% versus 31.7 ± 5.1%), which might correspond to the early immune response suggested by the MLR assay. However, the bone regeneration in allogeneic BMSCs was sustained throughout week 8 and 12. At week 8, the allogeneic BMSCs (61.8 ± 3.9%), autologous BMSCs (62.2 ± 0.1%) and cryogel alone (56.3 ± 5.2%) showed substantially more regeneration than the control group (35.9 ± 8.2%) (Figure 4G). At week 12, both allogeneic (91.3 ± 1.1%) and autologous BMSCs (93.5 ± 5.3%) showed statistically superior regeneration compared to the cryogel group (69.2 ± 2.7%) and control (42.50 ± 3.0) (Figure 4G). There was no statistical difference in bone regeneration between allogeneic and autologous BMSCs at week 8 or 12 (Figure 4G).

#### 3.4.2. Histology and Immunohistochemistry

**Figure 5:**
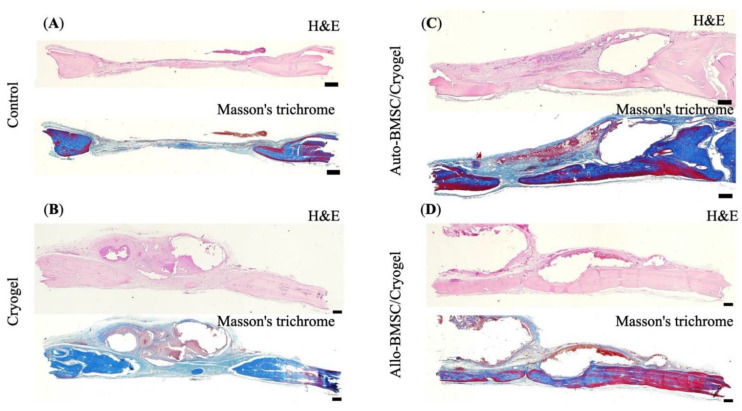
Histology of bone regenerative potentials at week 12. H&E stain and Masson’s Trichrome stain of (**A**) control (**B**) cryogel (**C**) autologous BMSC/cryogel (**D**) allogeneic BMSC/Cryogel, Scale bar = 500 μm.

**Figure 6:**
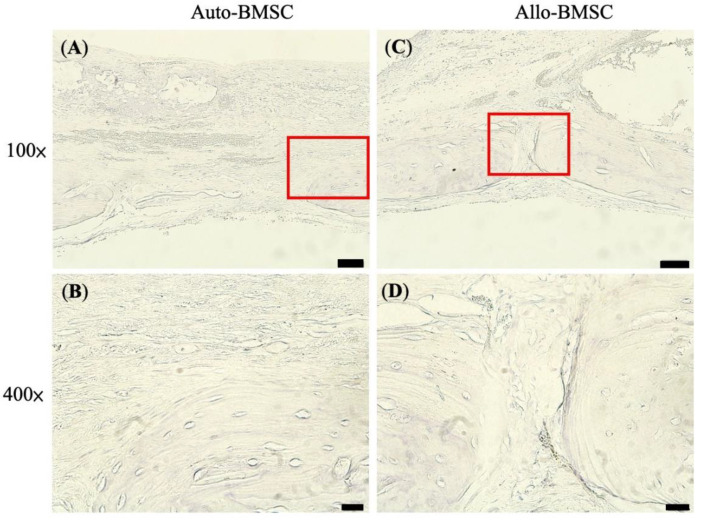
Immunohistochemistry of CD_4_^+^ T cells at week 12. Absence of CD_4_^+^ T cells in autologous BMSC-seeded cryogel (**A**) 100× (**B**) 400× and in allogeneic BMSC-seeded cryogel (**C**) 100× (**D**) 400×. Scale bar = 200 μm.

To reduce the total animal number in experiments, we continuously monitored bone regeneration by micro-CT. We euthanized rats for histology (H&E and Masson’s Trichromatic stains) and immunochemistry staining at week 12. Histologically, we revealed nearly a union of skull defects without remarkable lymphocyte infiltration in the allogeneic BMSC group (Figure 5G,H), which was comparable to autologous BMSCs (Figure 5E,F). In the control and cryogel groups, the defects were filled by fibrous tissue (Figure 5A–D). To further verify the immune response, we performed immunohistochemistry analysis using CD_4_^+^ marker because CD_4_^+^, but not CD_8_^+^, cells are responsible for the initiation of allogeneic transplant rejection [24,25]. We found no CD_4_^+^ lymphocyte infiltration along the regenerated bone at week 12 in either allogeneic or autologous BMSC group, suggesting the absence of CD_4_^+^-mediated immune response (Figure 6).

## 4. Discussion

Allogeneic BMSCs are a feasible solution in terms of bone regeneration when autologous BMSCs are not available. The rat cranium defect model verified that allogeneic BMSCs regenerated a comparable amount of bone defect compared to autologous BMSCs after 12-week inoculation in vivo.

Allogeneic BMSCs induced an early immune response, but bone regeneration was likely sustained by remaining BMSCs. Our MLR assay suggested a weak immune response at day 14, and micro-CT showed less regeneration compared to autologous BMSCs and cryogel alone at week 4, implicating an early immune response. (Figure 2E and Figure 3G) Nonetheless, we found that bone regeneration in allogeneic BMSCs continued after week four in vivo and became comparable with autologous BMSCs at week 8 and 12 via micro-CT. Theoretically, allogeneic BMSCs can establish immune tolerance by modulating innate and adaptive immunity [11,26]. This potential provides an explanation for our comparable results between allogeneic and autologous BMSC-seeded cryogels in bone regeneration.

Wu et al. also observed that the allogeneic BMSCs elicited an early (within 3 to 14 days) transient cell-mediated immune response with a surge of circulating IFN-γ, TNF-α, IL-2, CD_4_^+^ and CD_8_^+^ T cells compared to autologous BMSCs, but subsequently subsided to the same level after 14 to 28 days. Correspondingly, autologous BMSCs formed more osteoid than allogeneic BMSCs in the early stage but became comparable at later stages [11]. These results imply that the remaining cells may contribute to subsequent regeneration after escaping from acute rejection.

Outcomes of bone regeneration among allogeneic BMSC studies have been inconsistent, and a consensus of applying immunosuppressive modality or not is lacking. We summarize the literature in Table 1. In long bones, Arinzeh et al. and Guo et al. demonstrated that both allogeneic and autologous BMSCs could regenerate a defect without immunosuppression [10,12]. Guo et al. found constant bone regeneration through 12 weeks of monitoring by plain radiography [12]. In addition, the allogeneic BMSCs demonstrated equivalent osteogenic capacity to autologous BMSCs in mandible defects [11,13]. On the contrary, Rapp et al. found that the allogeneic BMSCs had less bone formation and impaired angiogenesis compared to autologous BMSCs in femur defects of humanized mice [27]. Rong et al. suggested that allogeneic MSCs required an immunosuppressive modality—by transfecting the surface ligands of B and T lymphocyte attenuator via Herpesvirus vector that inhibits the secretion of interleukin-17—to achieve adequate bone regeneration [14]. Coathup et al. further demonstrated that the allogeneic BMSCs failed to achieve bone regeneration, while autologous BMSCs demonstrated a superior osteogenic efficacy than osteoprogenitor cells [15]. Osteoclast-like cells accumulated and resorbed the bone around the defect in the allogeneic BMSC group, diminishing the extracortical bone regeneration. This evidence implies that immune rejection still plays a crucial role in the bone regeneration of allogeneic BMSCs.

Among the studies in Table 1, different bone models including femur, tibia, and mandible were discussed, and the defect size varied from 1mm to 50mm. The abovementioned literature used different animal models including canine, pig, ovine, and mouse. Besides, the choice of the scaffold was different in the studies including β-tricalcium phosphate, collagen type-I gel, demineralized bone matrix, or hydroxyapatite. Among all the studies, only Rong, Z., et al. used immunosuppressants to reduce the immune response of allogeneic BMSCs, and the suppressed group had better outcomes [14]. In addition, the result of osteogenesis by allogeneic BMSCs or autologous BMSCs had been inconsistent (Table 1). In our study, we created the calvarial defect on rats as our animal model with cryogel as a scaffold for BMSC implantation, in which the cyogel scaffold was different from the previous studies in Table 1. The difference of location of created bone defect and scaffold might explain both allogeneic and autologous BMSCs achieving 91.3 ± 1.1% and 93.5 ± 5.3% of bone healing at week 12. It is difficult to compare results between studies because of varied models, experimental species, cell number, and cell quality. However, the outcome can be maximized by selecting cells with appropriate characteristics. To further enrich the osteogenesis potential, we sorted for TNAP^+^ BMSC population to use in implantation. TNAP is considered a biomarker of osteoblasts. An increase in TNAP activity is detected in the initiation of osteogenic differentiation and positively correlates with osteogenic differentiation in BMSCs [28]. TNAP^+^ cytodeme also shows high expression of bone differentiation genes and low expression of cell cycle genes, implying an increased tendency toward bone differentiation [29].

The nHAP-gelatin cryogel scaffold used in this study comprises good affordability, biodegradability, and biocompatibility. Gelatin is an ideal scaffolding material that is highly similar in chemical composition to collagen. Along with nanohydroxyapatite (nHAP), which is similar to native HAP in bone and possesses excellent biocompatibility and osteoconductive properties, a 3D-designed gelatin/nanohydroxyapatite (nHAP) cryogel with cross-linking agents 1-ethyl-3-(3-dimethylaminopropyl) carbodiimide (EDC) enhanced osteogenesis efficacy of BMSCs. The cryogel is also characterized by superior plasticity and tailoring, suitable for use in regions such as the craniofacial area [20]. Rodrigues et al. demonstrated an increase in cell proliferation of MG63, the osteoblast-like cells, in collagen-nHAP cryogel scaffold, suggesting an osteoconduction effect of nHAP [30]. Previous studies have demonstrated the potential of cryogel scaffold use in tissue engineering of bone [21,31,32], adipose tissue [33], and cartilage [34]. With the facilitation of nHAP-gelatin cryogel scaffold, both allogeneic and autologous BMSCs can have more dedicated effects toward bone regeneration.

Like direct BMSC implantation in bone defects, ectopic transplantation of BMSCs with a scaffold will also elicit an early immune reaction. Chatterjea et al. performed ectopic implantation of autologous and allogeneic osteoprogenitor cells in dorsal subcutaneous pockets of mice, and the allogeneic constructions showed a strongly positive T cell and B cell reaction. This immune reaction precludes allogeneic bone formation despite administrating adequate immunosuppressants [17]. Wu et al. also performed ectopic subcutaneous implantation of autologous and allogeneic BMSC-generated bone on beagle dogs, in which the ELISA immunoassay revealed a significantly higher IL-2 level and lower IL-10 level in the allogeneic BMSC group at an early stage, but subsequently reached similar levels to autologous BMSCs after 56–84 days [11]. The bone formation was significantly higher in autologous groups within 8 weeks but reached the same level by week 12 among groups. The allogeneic BMSCs elicited markedly prolonged immune reaction when implanted subcutaneously than in bone, indicating different immunological niche in allogeneic bone regeneration.

The detailed mechanism of bone regeneration by allogeneic BMSCs is not yet fully elucidated, either from differentiation of the implanted cells or the paracrine effects [35,36,37]. There has been some evidence that provides possible explanations.

Angiogenesis as well as osteogenesis plays crucial roles concerning bone regeneration and formation. Wu et al. proved that via stimulating exosomal miR-1260a secretion, exosomes derived from BMSCs could promote both osteogenesis and angiogenesis by suppressing HDAC7 and COL4A2 expression [38]. Lu et al. also proved that miR-29a promoting angiogenesis and osteogenesis in vivo [39]. Zhao identified macrophage scavenger receptor 1 (MSR1)) mediated PI3K/AKT/GSK3β/β-catenin signaling, which promoted osteogenic differentiation of BMSCs [40].

In our study, the mechanisms of gelatin-nanohydroxyapatite cryogel as a scaffold with BMSCs is hypothesized that the cryogel could have a shield effect on the cell inside, leading to a postponed immune attack and can prolong the paracrine effect. A serial histology section examination will be scheduled in our following experiments.

There are some limitations to the current study. We aimed to verify the outcome of bone regeneration and compare the final osteogenic efficacy among allogeneic and autologous BMSCs. Although we evaluated the immune response in vivo at week 12, it may not comprehensively reflect the immune responses that occurred throughout the regeneration process. To further elucidate the status of early rejection and immune tolerance, a study of the comprehensive interplay between allogeneic cells, scaffold, and the immune system is required.

## 5. Conclusions

Our study demonstrated that allogeneic BMSC-seeded cryogel scaffolds could effectively induce bone regeneration in calvarial defects after 12 weeks of implantation, comparable to autologous BMSC-seeded cryogel scaffolds. Further, despite a transient early immune response in allogeneic transplantation, bone regeneration was sustained throughout the 12-week implantation period, with the CD_4_^+^-mediated immune response diminished by week 12. These results suggest that allogeneic BSMCs are an effective alternative cell source in craniofacial bone regeneration and warrant further investigation for future clinical applications.

## Figures and Tables

**Table 1:** Allogeneic mesenchymal stem cells in bone regeneration.

Long Bone						
Reference	Animal Model	Cells	Scaffold	Immunosuppressants	In Vitro Results	In Vivo Results
Arinzeh, T.L. et al., 2003 [10]	Canine Femoral diaphysis mid-portion 21mm defect.	Allogeneic BMSC Autologous BMSC	HA-TCP	n/a	MLR: Significant T cell proliferation	16 Weeks Allogeneic BMSC (49%) = Autologous BMSC (42%) > HA-TCP (24%)
Guo, S.Q. et al., 2009 [12]	Pig Middle tibia shaft 20mm Segmental defects	Allogeneic BMSC Autologous BMSC	β-TCP	n/a	MLR: SI not significant increase	++ 16 Weeks Allogeneic BMSC (75%) = Autologous BMSC (75%) > β-TCP (42%) > blank (7%)
Coathup, M.J. et al., 2012 [15]	Ovine Tibial bone defect 50mm Prosthesis inserted	Allogeneic BMSC Autologous BMSC OPC	HA	n/a	MLR: Significant T cell proliferation	6 months Autologous BMSC (149.5 mm^2^) > OPC (121.1 mm^2^) > Control (87.5 mm^2^) > Allogeneic BMSC (0 mm^2^)
Rong, Z. et al., 2017 [14]	Mouse Femur 1mm defect	Allogeneic BMSC Allogeneic HVEM-expressing BMSC	DBM	HVEM transfection	Allogeneic HVEM-expressing BMSC inhibit IL-17 secretion	8 weeks Allogeneic HVEM-expressing BMSCs (73%) > Allogeneic BMSC (39%) > DBM (15%)
Rapp A.E. et al., 2018 [26]	Humanized Mouse Femur1mm defect	Allogeneic BMSC Autologous BMSC	Collagen type-I gel	n/a	n/a	35 days Autologous BMSC (37%) > Allogeneic BMSC (16%)
Craniofacial bone						
De Kok, I.J. et al., 2003 [13]	Beagle dog Bilateral mandible alveolar bone 6.5 × 20 mm	Allogeneic BMSC Autologous BMSC	HA-TCP	n/a	MLR: Significant T cell proliferation	9 weeks Allogeneic BMSC (85%) > Autologous BMSC (83%)
Wu, J. et al., 2016 [11]	Beagle dog Mandibular body 30mm defect	Allogeneic BMSC Autologous BMSC	β-TCP	n/a	MLR: SI significant increase	24 weeks Auto-bone (82%) > Allogeneic BMSC (44%) > Autologous BMSC (39%)

HA-TCP: hydroxyapatite-tricalcium phosphate; MLR: mixed lymphocyte reaction; MHC: major histocompatibility complex; β-TCP: β-tricalcium phosphate; SI: stimulation index; OPC: osteoprogenitor cell; HA: hydroxyapatite; DBM: demineralized bone matrix; HVEM: herpesvirus-entry mediator. ++ Bone healing score.

## Data Availability

No new data were created or analyzed in this study. Data sharing is not applicable to this article.
